# When war came home: air-raid shock in World War I

**DOI:** 10.1177/0957154X21998217

**Published:** 2021-03-09

**Authors:** Stefanie Caroline Linden

**Affiliations:** Maastricht University, The Netherlands

**Keywords:** Britain, case records, psychiatry, shell shock, trauma, war medicine, 20th century

## Abstract

During World War I, civilians became a target of the war machine. Air raids transformed the lives of those not involved in active combat and blurred the lines between the home front and the war front. This paper argues that the experience of air raids in World War I was comparable to the combat stress at the Western Front. The author bases her argument on contemporary publications in medical journals, measures taken by British authorities to prevent air-raid shock, and contemporary case records. The narratives of air-raid shock – similarly to those of shell-shocked soldiers – reflect the feelings of terror and loss of control, and demonstrate the profound effect these experiences could have on individuals’ mental health.

## Introduction

The psychological trauma of World War I has been inextricably linked to combat-related mental breakdown in soldiers, often subsumed under the heading of ‘shell shock’. With the centenary of World War I, much has been written about shell shock and the devastating consequences of war for the psychological health of soldiers. However, the theatre of war reached far beyond the battlefields of France and Flanders. For the first time in history, the idea that the home front and the war front were naturally separated was challenged by the technological changes that accompanied World War I, particularly the use of Zeppelins and planes to attack civilian populations (Grayzel, 2006: 590). The changes also threatened to blur the traditional distinction between soldier and civilian and awakened a ‘new sense of vulnerability’ (Robb, 2015: 22). With this expansion of warfare into civilian spaces, women, children and non-combatant men became targets of the war machine.

Susan Grayzel was the first to focus on the impact of air raids on the civilian population in World War I. In *Women’s Identities at War*, Grayzel (1999) described how technological advances changed the nature of warfare during World War I, leading to a ‘breakdown of meaningful distinctions between home front and front line’ (p. 45). However, she rejected the idea that traumatic events on the home front could have the same transformative effects on a person’s mental health as combat stress on the soldier. Based on reports in the contemporary press, Grayzel emphasized women’s and children’s resilience and their mental strength in the face of the new threat from the skies. Referring to a case of infanticide, she questioned the causal link between air raids and mental breakdown (pp. 46–8).^1^

Similarly, focusing on air raids in France in World War I, Grayzel (2006) used narratives of physical injury and death but did not expand on psychological casualties of the air raids. She concluded that civilians in France coped relatively well with the new threat and that the expansion of German warfare did not lead to the intended intimidation of the civilian population, but only enhanced the outrage over German cruelty and immorality.

In her most recent and most comprehensive monograph, *At Home and Under Fire: Air Raids and Culture in Britain from the Great War to the Blitz*, Grayzel (2012) analysed responses to air raids in the media, government pronouncements and popular culture, as well as first-hand accounts of aerial warfare in letters and diaries. She closed an important gap in the historiography of World War I by dedicating the main part of her book to the consequences of air warfare for the civilian population. She described how air raids and the attack on ‘innocent’ civilian populations stirred anti-German feelings, encouraged acts of bravery and boosted recruitment of volunteers (pp. 37, 68). Grayzel argued that ‘the experience of aerial warfare forms a crucial aspect of the wartime narrative’ and dominates civilian memoirs long after the war (p. 95).

Grayzel’s analysis of wartime narratives demonstrates the wide range of human reactions to the new threat of aerial warfare, from anger to fear, terror to excitement. Although she illustrates the enormous strain and suffering imposed on the civilian population, she left one topic largely untouched: the enduring effects of air warfare on the mental health of civilians. According to Grayzel (p. 92), ‘air raids were domesticated and normalized in daily life by the end of the war’ and ‘had become part of the lived experience’. In that sense, air raids would have been fundamentally different from the combat experience of the soldiers at the front line, which continued to trigger severe mental trauma up to the end of the war and beyond (Linden, 2017). It seems surprising that such a fundamental difference should have existed, given the many similarities between the combat and air-raid trauma (such as imminent threat to life, experience of casualties of comrades/family members, extreme loss of control), and indeed I will argue in this paper that air raids, too, could have transformative and enduring impact on mental health and triggered syndromes that were almost indistinguishable from shell shock. I will base my argument on three lines of evidence: first, the contemporary literature (both lay and professional) attesting to psychological and psychiatric consequences of air-raid shock; second, the measures taken by British authorities to cater for victims of air-raid trauma, particularly children; and third, the case records of air-raid patients of the National Hospital for the Paralysed and Epileptic at Queen Square in London. I will conclude with an analysis of the aftermath of the debate on air-raid shock, which charts the shift from psychological to organic diagnoses and also traces how the existence of air-raid shock fell into near-oblivion in the inter-war years, apart from a revival before World War II.

## World War I and civilians: the impact on everyday life

We say, that happened before the war, during the war, after the war, and it is not strange that we should measure time thus, for the war cut our life into three periods. . . . in the course of a few weeks we were forced into ways of life utterly strange to us, from which four years later we emerged in the distracted fashion of creatures whose shelter has been removed . . . . (Peel, 1929: 1)

In her 1929 monograph *How We Lived Then*, Herefordshire-born journalist and writer Constance Peel (1868–1934) described how, from the very beginning of the war, everyday life was transformed and the civilian population suffered increasing hardship. Many homes were broken up by the departure of the troops and the calling up of reserves. Already in the first months of the war, the civilian population battled with increasing prices and shortages of coal, food and medication (pp. 55–6). Many women dedicated their time to charitable work in nursing and performing domestic duties in military hospitals, and gradually they replaced all men who had been called up; they took over typically male occupations and many did extremely dangerous work, for example in explosives and shell-filling factories. In addition to the struggles of domestic life, civilians were continuously reminded of the horrors of fighting by the tide of wounded men pouring into the home hospitals. Access to medical care became increasingly difficult because of the shortages of medical staff, overcrowded hospitals and the long waiting lists (Linden, Jones and Lees, 2013).

Due to the wartime shortages and the anticipated aerial attacks, the whole atmosphere of the towns and cities changed; from the autumn of 1914 the street lamps were dimmed and painted black on top, and no big groups or long lines of light were permitted. Everyone had to use blinds to block their windows at night. ‘London had gone back twenty years as regards lighting. By the end of the war, it was almost as dark in the streets as it had been in the Middle Ages’ (Peel, 1929: 55).

However, as Peel shows, ‘the events which brought war home to us in more senses than one were the bombardment of coast towns by war vessels and the raids of airships and aeroplanes’ (p. 138). With the first bombardment on 16 December 1914, which targeted the coastal towns of Hartlepool, Scarborough and Whitby, came the realization that war had arrived in England.

## Air raids on Britain

At the beginning of the war, the Germans used Zeppelins for reconnaissance purposes only, but in January 1915 Emperor Wilhelm approved the aerial bombardment of military targets such as coastal defences and the London Docks (Fegan, 2002: 16). The British mourned their first ever air-raid casualties on 19 January 1915 in Great Yarmouth where two people were killed. The British press denounced the attack as an act of cowardice and downplayed its military significance (p. 19). Similarly, in a letter to the editor of *The Times*, a reader questioned the military purpose of the raid and stated that the

sole intention was to inflict injury on non-combatants in order to create a panic . . . and to frighten the Government of the country in which they live . . . . We hold in this country that attacks of this kind are contrary alike to war and to humanity and that they cannot be defended on any conceivable ground. (Richards, 1915)

The first German Zeppelin attack on London happened on 31 May 1915. Seven people were killed in the East End – among them four children – and 35 were wounded. Londoners were outraged, and Germans living in the capital were attacked and their businesses destroyed. More Zeppelin attacks on London continued for the rest of 1915 and most of 1916. On 31 September 1916, the first Zeppelin was shot down close to Margate. Afterwards, several airships were brought down, for example the SL11 at Cuffley in Hertfordshire on 3 September 1916 and two airships on 27–28 November 1916 off the coast at Hartlepool. British air defence had been successful, and by the end of 1916 over 17,000 officers and other ranks were devoted to air defence, anti-aircraft batteries and searchlights, and 110 aeroplanes formed the Home Defence squadrons (Fegan, 2002: 40).

The German Army, disillusioned by these defeats, abandoned the airships and prepared for attacking Britain with aeroplanes. The Germans had considered using planes for the bombing of Britain from the early days of the war and their planes had attacked the south-east coast from occupied Belgium on 21 December 1914, a month before the Zeppelin campaign was launched, but the systematic use of planes had been hampered by their limited range and restricted bomb capacity. Gotha bombers – which could tackle longer distances and carry heavier bombs – were used to attack civilian populations from May 1917. They had many advantages over the Zeppelins: they were fast (up to 80 mph), could be navigated more easily and were less vulnerable to incendiary bullets than the hydrogen-filled Zeppelins; they were also less visible and less likely to be detected by British Air Defence (Fegan, 2002: 48–9).^2^ The introduction of these planes meant that the British population could be attacked more efficiently – and during daylight hours. The deadliest air raid of the war took place during daylight on 13 June 1917, killing more than 150 civilians and injuring more than 400 in Margate, Essex, and in London (Peel, 1929: 147–8; Robb, 2015: 21).^3^ The bombing of a London County Council school in Poplar caused most outrage as it killed 18 children, of whom 16 were aged between 4 and 6 years, and injured more than 30 others.

The authorities rushed to develop an elaborate air defence of warning sirens, searchlights, anti-aircraft guns and barrage balloons. On 22 July 1917, maroons (sound bombs used as distress signals at sea) were fired in London to alert the public for the first time. To protect the inhabitants of the capital against night raids, Londoners could take refuge in the Underground stations. Those who could afford it left the city and went to places to which the Zeppelins and aeroplanes did not penetrate, such as Bath and Bournemouth (Peel, 1929: 154). Children were sent away, or kept home from school.

Over the war years, 1413 people were killed and 1972 wounded as a result of German air raids. Although the numbers of these casualties were small in comparison with those on the battlefields of the Western Front, the psychological impact of the bombings was huge (Robb, 2015: 22).

## Mental distress caused by the air raids: the contemporary evidence

Air raids on Britain had been anticipated in fictional accounts of future wars and had preoccupied popular imagination for several decades before the war (Fegan, 2002: 10). However, the population was already suffering from the consequences of war, and mental distress and restlessness were observed within the families whose husbands and fathers had been called for service. Peel (1929: 61) reported that the ‘mental strain, the desire to forget horrors and unhappiness, led to an increase of drinking, drugging, smoking, gambling and dancing’. The air raids thus came as an additional source of terror for an already distressed civilian population. On analysing Londoners’ reactions to air raids in World War I, White (2014: 126) describes an ‘unpredictable mixture of sangfroid and blind terror’. Indeed, the emotional response of the British people was variable, oscillating between both extremes, and the press naturally indulged in stories of bravery and stoicism. Some personal letters attest to feelings of panic and despair (Grayzel, 2012: 76–7). However, when scrutinizing the whole available evidence, three emotional reactions to aerial attacks seem to have dominated.

**Figure 1.**
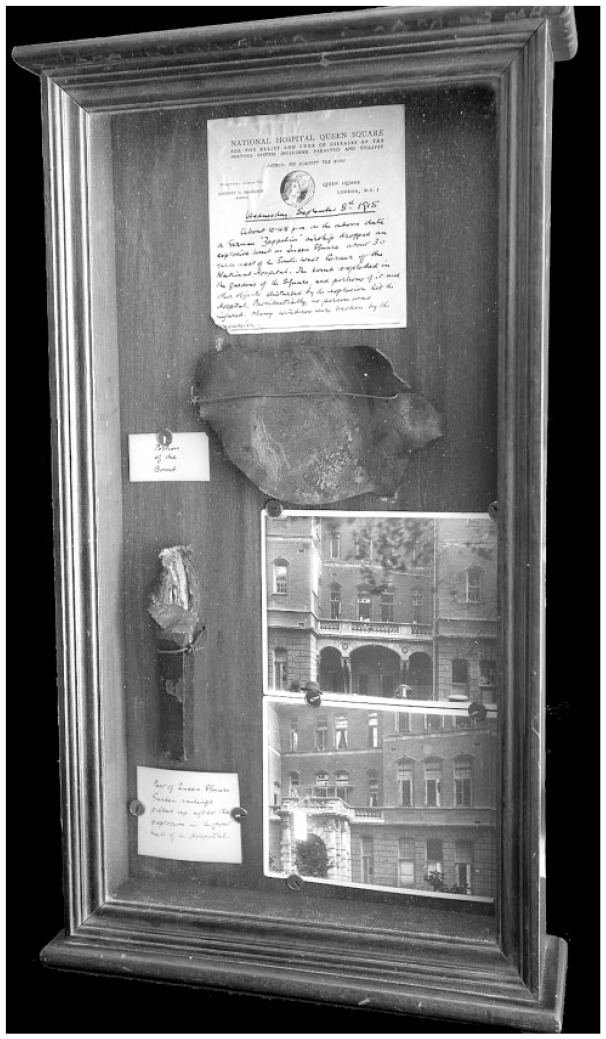
Cabinet containing evidence of bomb damage in Queen Square from a Zeppelin raid in September 1915, including a note describing the event, photos, part of the garden railing and a fragment of the bomb (QSA/15424; Queen Square Library, Archive and Museum).

The first frequently described response was fascination and excitement. People were spellbound by the giant emergence from the sky. Peel (1929: 144) described the challenges of making people take shelter because ‘they would rush into the streets and stand gazing up the intruder’. This fascination was also shown by the thousands of people who visited – and paid to visit – the sites of destruction after the Zeppelin raids, or travelled to view the wreckages of Zeppelins that had been shot down by the British, for example at Cuffley (see previous section), where visitors collected pieces of wire and charred wood as souvenirs (Fegan, 2002: 32). The section below on medical case records at the National Hospital, in Queen Square in London, starts with a description of a Zeppelin raid there. Afterwards, the hospital staff collected material from the bombs and parts of the damaged garden fence and put them into a memorial shrine which can still be viewed today (see Figure 1).

The second emotional response was anger. After the first raid on London, German citizens were intimidated and attacked and their businesses vandalized. When the airship was shot down at Cuffley, the local vicar refused to bury the German crew at his church. When the German airmen were eventually buried with full military honours near Potters Bar (a town 4 miles west of Cuffley), there were angry crowds, and one woman ‘hurle[d] eggs at their coffins’. Lieutenant William Leefe Robinson, the 19-year-old who shot down the airship, became a national hero and was awarded the Victoria Cross, the only one presented for an action in Britain (Fegan, 2002: 34).

Finally, the third response was one of terror and fright. When the air raids started, some ‘of the less controlled screamed and cried, frightening the children, who otherwise might have looked upon the affair as a pleasant variation from the usual routine’ (Peel, 1929: 154). Even air-raid warnings could cause panic and unrest (Grayzel, 2012: 79).^4^ Along similar lines, Fegan (2002: 45) noted that ‘the dread caused by the threat of raids had more effect on people’s daily lives and war production than the actual bombs that were dropped’.

In his article on the mental aspects of air raid precautions, the wartime physician John Rickman – looking back at his experiences of World War I – described four stages in the reaction of ‘normal people’ to acute war danger, in particular in reaction to air raids (Rickman, 1939). The initial reaction to the threat was characterized by ‘the feeling that one will not be able to maintain self-control much longer’. This state of anxiety was commonly transient, and people regained self-control within a short period of time. If the danger persisted and/or no reassurance or ‘moral reinforcement’ was given, some people progressed to the ‘acute stage’ which was characterized by increased or decreased motor activity, the latter sometimes leading to stupor, the complete cessation of movements. According to Rickman, individuals in this state were still accessible to reassurance. However, failing this support, people with a ‘predisposition to nervous states’ could develop real panic, a state of complete loss of self-control in which ‘all social ties were temporarily severed’ (p. 457). Rickman emphasized that only those people with latent neurotic conflicts which originated in infancy were in real danger of breaking down under these circumstances. Even if people progressed to this state of disarray, they naturally regained control of the situation, resuming their social contacts, responding to commands and realizing their responsibilities. We will see later, when reviewing the case records, that this latter assessment was too optimistic, at least for some of those who suffered mental breakdown as a consequence of the air raids.

Publications on psychological consequences of air raids in medical journals during the war years were sparse. One 1917 article on ‘Air raid psychology’ published in *The Lancet* discussed individual and mass reactions to aerial bombardments (Anon., 1917a). The author argued that, whereas individuals adapted and managed to stay in control of the situation, crowds were less predictable and tended to be ‘impulsive, mobile, unstable’. It was also suggested that the novelty of the air-raid experience and the lack of adaptation to this new threat explained why some individuals or groups initially reacted in an instinctive and often disordered and erratic way.

The more unusual, strange, unfamiliar the exciting stimulus, the more overpowering is the instinct likely to prove – Omne ignotum pro magnifico^5^ is a very old saying, but also a very true one. When, therefore, without warning, a bomb drops from the blue in a crowded thoroughfare to dash for the nearest shelter is as instinctive an act as for the puppy to hunt the first rabbit that crosses its path. Further, what is strange also excites the instinct of curiosity, which, once aroused, may come into conflict with the other instinct; . . . With increasing knowledge our behaviour becomes more and more rational . . . . (Anon., 1917a)

The author acknowledged that fear was a natural response to the air-raid warnings and the approaching invaders. He stressed the similarities between nervous reactions to air raids, and the ‘shell shock’ of the firing line which could both be characterised by ‘fits, hysterical and epileptic, . . . strokes of paralysis from excitement and heart failures’. However, the author argued that individuals quickly adapted to the new situation, overcame their fear and became rational in their future responses to the same threat. Analysing the reaction to air warfare, the author praised British resilience, calmness and ‘exemplary conduct’ and concluded that extreme psychological reactions to the air raids ‘have not been common occurrences’. Along the same lines, another article in *The Lancet* praised the ‘growing bravery . . . which accounts for the fact that a short relief from strain is almost invariably followed by a complete recovery of balance’ (Anon., 1917c).

A further report in *The Lancet* provided convincing evidence that even the most vulnerable individuals – in-patients of various London hospitals – remained calm when facing repeated aerial attacks (Anon., 1917b). The inquiries made at various institutions in London yielded responses such as ‘the calmness of the patients was wonderful’ and ‘patients admitted immediately after the raid showed no signs of panic’, but there were also anecdotes of individual panic and unrest. At Guy’s Hospital one man who had his leg amputated suffered a shock and ‘cried like a child for four or five hours’. At St Thomas’s Hospital, some of the bedridden soldiers were nervous; and at the London Fever Hospital, one patient ‘took refuge under her bed and another in a coat-cupboard’. Other patients reacted with anger, for example ‘the boys with scarlet fever’ who ‘took to active vocal verbalisms such as ‘fat-heads’, ‘square-heads’ and ‘pigs of Germans’.

Contemporary publications mirror the particular concern for women’s mental stability, and the potential adverse effects that air raids and air-raid warnings might have on their psychological health. However, during the war years the media consistently reported women’s resilience and the absence of panic. Ryan (1916)^6^ emphasized that:

one waits in vain for descriptions of the hysterical women, the woman who shrieked with fear or the woman who fainted. There are no such descriptions because there were no such women. The most extraordinary feature of each of the recent raids has been the calm with which they were faced by women and children.

There seemed to be a general consensus that civilian reactions to air raids were transient and well in proportion to the novel, unexpected threat. However, this picture of strength, stamina and resilience was challenged by some emerging evidence of mental breakdown within the civilian population, and also by air-raid-related admissions to one of the leading London hospitals.

## Functional disorders and air raids

The vast majority of articles in medical journals and in the press reported the physical injuries inflicted by Zeppelin and aeroplane bombs (Wells, 1916) and denied mass panic and mental breakdown. Anxiety and restlessness were described as transient self-limiting reactions. Others mentioned psychiatric casualties, but remained vague and did not provide any numbers or specific symptoms.

However, it has to be considered that the British Government had imposed censorship on the reporting of air raids to prevent the Germans finding out how effective and accurate their attacks had been, and to keep the public calm. So ‘the government persisted with its official line that the raids were insignificant and the newspapers faithfully portrayed the British as a cheerful and plucky lot, calm and unworried by the German air menace’ (Fegan, 2002: 41).

After the war, more evidence of civilian trauma due to the air-raid experience seemed to emerge. For example, in her post-war memoir, Peel (1929: 147–8) describes ‘many cases of nerve trouble more or less serious and enduring which resulted from some of the horrible experiences to which raid victims were exposed’. When the discussion on psychological consequences of air raids was revived in expectation of a new European war, it became obvious that there was a real concern that severe psychiatric casualties could affect civilian morale and mental stamina in a future war. Based on the experiences of World War I, Maurice Wright (1939) attempted a classification of air-raid-related psychiatric casualties. In anxiety hysteria, simple terror had progressed into a pathological reaction, preventing people from reacting rationally:

Severe cases of anxiety hysteria . . . may be brought in from the streets or air raid shelters, collapsed and tremulous, or wandering in a purposeless way with clouded consciousness or even amnesia; near to any severe explosion, they may be found lying apparently unconscious in the state of hysterical coma or stupor . . . with a constant expression of terror, a coarse tremor, sweating, and tachycardia; they scream sometimes if touched, and some lie curled up under the bedclothes in the intra-uterine position. . . . Evacuation is imperative, because if these cases are sent home, they will certainly relapse and become sources of infection to those around them who may be themselves on the verge of breakdown. (p. 577)

It is striking how much this description of air-raid shock resembles the reaction of soldiers traumatized during active combat at the Western Front. Soldiers also developed so-called dissociative reactions in which they were in an altered state of consciousness, sometimes unresponsive and paralysed with fear. Some regressed to child-like behaviours, others developed striking physical reactions such as trembling or shaking. These acute reactions could progress into conversion hysteria in which anxiety and fear were unconsciously converted into physical symptoms, both in shell-shocked soldiers and in civilians traumatized by air raids. Wright also describes other psychiatric presentations following the air raids, such as psychoses and hysterical amnesias. Wright – based on his experience from World War I – anticipated an uncertain number of psychiatric casualties as a result of a frequent and intense bombardment of the civilian population. He suggested putting rigorous protective measures in place ‘before the emergency arises’ to boost civilian morale and prevent collective mental breakdown.

## Medical case records and air-raid shock: cases at the National Hospital

The strongest evidence that air raids could have a transformative and enduring impact on civilians’ mental health and could trigger clinical symptoms that were almost indistinguishable from shell shock comes from medical case records. Not many records have survived, but those from the National Hospital for the Paralysed and Epileptic (hereafter NH) in London are complete and cover all admissions from World War I and the post-war period.

**Figure 2.**
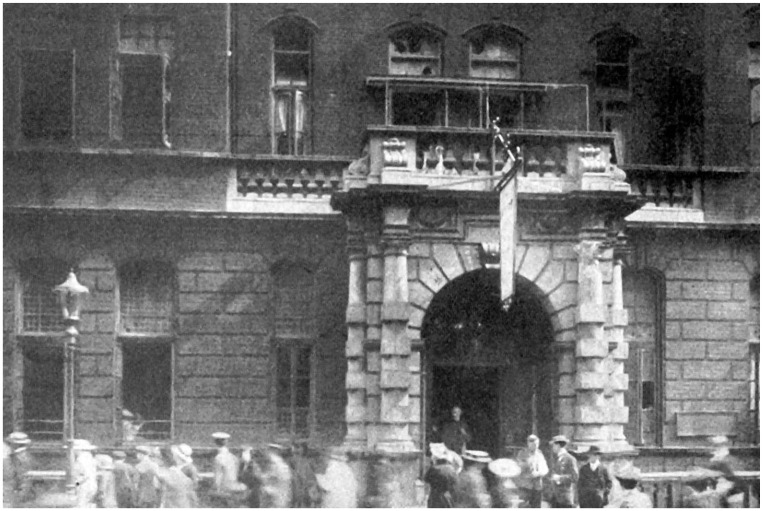
Hospital on 8 September 1915 after the Zeppelin raid (QSA/19210; Queen Square Library, Archive and Museum).

Before the outbreak of World War I, this hospital, in Queen Square in the heart of London, had already gained an international reputation for the treatment of neurological conditions, and had pioneered neurosurgery in Britain. Soon after the beginning of the war, soldiers with head wounds, gun-shot injuries and traumatic nerve lesions were treated at the hospital, along with hundreds of shell-shocked soldiers (Linden et al., 2013). The hospital buildings did not escape the German war machine (see Figure 2): on Wednesday 8 September 1915,

about 10.45 pm . . . a German ‘Zeppelin’ airship [had] dropped an explosive bomb in Queen Square about 30 yards west of the South West Corner of the National Hospital. The bomb exploded in the gardens of the Square and portions of it and other objects disturbed by the explosion hit the Hospital . . . . Many windows were broken by the concussion. (Source: Handwritten note in cabinet shown in Figure 1)

Several inpatients – well-off ladies with hysterical paralyses who had been bedridden – ‘rushed from their beds and were later found scattered through the hospital’ (Holmes, 1954: 58).

The bombing came just a month after the hospital’s Board of Management had resolved to insure the hospital against aircraft risks. Civilian patients who were treated on the same wards as the shell-shocked soldiers provided a harrowing account of what it was like to live in a city at war. The lives of some of them had been profoundly transformed by the air-raid experience of the capital. The present paper focuses on all civilian patients with functional (neurological and somatic) symptoms treated in the NH between 1914 and 1924 whose symptoms were either triggered or exacerbated by the air-raids.

## Source material and analysis

The Queen Square Archives hold 1500 volumes of case notes, the longest continuous manuscript collection of neurological case histories in Britain (Etherton, 2011), with patients being admitted from across Britain and, during the war, from all the Allied countries. The Queen Square records contain basic sociodemographic data (date of admission/discharge, occupation, marital status, nationality, date of birth, address, sex), diagnosis, name of consultant and Responsible Medical Officer (RMO), as well as accounts of symptoms, family history, past medical history, drug/alcohol and social history, therapies and treatment outcomes. Physical (including neurological) examinations are documented in a very detailed and standardized form. The records also provide follow-up notes, daily monitoring charts, diet and medicine charts. Every record starts with a detailed patient narrative, which generally covers his or her account of the development of symptoms, the triggering events, the patient’s own explanation of symptoms, and his or her journey through the medical system. Approximately 40% of case notes are handwritten and 60% are typewritten. The author read all cases from 1914–24 (about 10,000 records) and took digital photographs of the relevant cases. This study includes all patients with functional disorders, admitted between 3 August 1914 (the date of the British entry into the war) and the end of 1924, whose onset of symptoms was clearly related to air raids. It deals only with civilian cases (not soldiers who also broke down after air raids), and the symptoms had to be unrelated to a neurological/medical condition (functional disorders) and either directly triggered or exacerbated by air raids.

From the records, the author created an SPSS datasheet (version 25.0., IBM, Armonk, NY, USA) with sociodemographic and other key variables, including diagnostic labels, symptoms and treatments.

### Sociodemographic data

The author identified 58 cases (51 adults, 7 children) of air-raid shock in the war and post-war years. The sociodemographic characteristics of the sample are described in Table 1. Most patients admitted with functional disorders caused or triggered by air raids were female (43 female, 15 male admissions) and belonged to the lower middle class (62.1%). Most adult patients were married (*n* = 29, 56.9%). The average age was 34.5 (±15.5) years; the youngest patient with air-raid shock was 8 and the oldest 63 years old.

**Table 1.** Sociodemographic characteristics of all cases of civilians with air-raid shock (*N* = 58).

Age on admission in years:	
mean (±SD)†	34.5 (±15.5)
[range]	[8–63]
Gender (m/f)	15/43
Adults	51
Children (<16 years)	7
Nationality	
British	54
Belgian	1
Polish	1
Swiss	1
Dutch	1
Admissions per year	
1915	4
1916	2
1917	9
1918	3
1919	13
1920	5
1921	5
1922	7
1923	5
1924	5
Length of stay in days:	
mean (±SD)^†^	58.6 (±36.4)
[range]	[2–175]
Marital status (of adults)	
Single	22 (43.1%)
Married/widowed	29 (56.9%)
Social class*	
Unskilled workers	15 (25.9%)
Lower middle class	36 (62.1%)
Upper middle class	4 (6.9%)
Not known	3 (5.2%)
Diagnosis	
Hysteria	9 (15.5%)
Neurasthenia	14 (24.1%)
Neurosis	2 (3.4%)
Functional disorder	3 (5.2%)
No diagnostic judgement	4 (6.9%)
Psychiatric diagnosis**	11 (19.0%)
Organic diagnosis, no evidence for organic disease***	15 (25.9%)
Trigger	
Air raid as primary trigger	42
Symptoms exacerbated by air raid	16
Treatment	
Sedatives only	16 (27.6%)
Electrotherapy	15 (25.9%)
Isolation	3 (5.2%)
Physical	30 (51.7%)
Exercise	12 (20.7%)
Persuasion	2 (5.2%)
Glandular extract (polyglandin)	1 (1.7%)
Treatment result	
Cured	6 (12.1%)
Improved	35 (60.3%)
In status quo/worse	17 (29.3%)

† Standard deviation.

* Unskilled workers: e.g. labourers, maids, miners and farm workers; lower middle class includes craftsmen and owners of small businesses; upper middle class: physicians, teachers and clergy. Upper-class patients were defined as people with independent sources of income. Percentages in brackets reflect the proportion of cases belonging to one class in relation to those cases in which the social class was known.

** Depression (2), dementia praecox (3), delusional insanity (1), tic (5, at the time seen as psychological/psychiatric condition).

*** Disseminated sclerosis (8), epilepsy (3), toxic neuritis (1), diabetes (1), tetany (1), Meniere’s disease (1, seen as organic condition).

### The cases

The admissions for air-raid shock sparked less interest among clinicians than the shell-shock cases (Linden and Jones, 2014), as indicated by the less detailed case histories and documentations within the medical case records. Unlike the shell-shock cases, the cases of air-raid shock are often ‘disguised’ as organic conditions, and the link to the air raids only becomes obvious when reading the history (rather than just the cover page of the record). Some patients were treated at the NH during the time of the raids on London, for example Edith G, a 30-year-old clerk from London who was an inpatient at the hospital from 29 June to 20 July 1917. She had been suffering from trembling of her hands and legs for almost two years following the London Zeppelin raids in October 1915:

October 1915 during the Zeppelin raid bombs dropped within 100 yds of her about 9.15pm, at the time she was walking with her brother. She was badly frightened, did not fall but shook all over. Shakiness progressively got worse but she continued working in the office until June 1916 when she was obliged to take a holiday on account of the unsteadiness of her hands and legs. The legs became weak, she was unable to walk. She recovered from the paralysis of the lower limbs about the end of October 1916. She was in fairly good condition until February 1917 when she began to shake again. She returned to work in October 1916 and was in fairly good condition until March 1st when she was ordered to Folkestone for 3 months. She was in Folkestone 5 weeks, getting on fairly well until the raid there. She again became upset and the use of the lower limbs went, she returned home for 3 weeks and has been waiting to come in here. In November 1916 her voice left her but returned in about 4 days. (Edith G, QSR, 1917)

Edith G also suffered from fainting attacks in which she lost consciousness, ‘gnawing pain’ in her chest and ‘dull aching pain’ in the frontal region of her head. On examination, the doctors also found ‘glove and stocking anaesthesia’, a loss of feeling in her hands and feet. All her symptoms, the trembling of hands and legs, loss of speech and paralysis of her legs, had been triggered by air raids. Lewis Ralph Yealland, the renowned shell shock doctor (Linden et al., 2013), treated her with ‘gentle faradism to the arms’ and also with ‘psychotherapy’. After one treatment session, Edith G was cured.

On 21 August 1917, one month after Edith’s discharge, 18-year-old May G from Scotland was admitted to the NH with a six-month history of ‘hysterical tremor’ of her left arm and weakness of the left leg. The case record reads:

Tremor of the left arm appeared suddenly one evening about 9pm. She was sitting down reading a book at the time and suddenly became fearful of an air raid. Her mother was in the room at the time, patient was not talking to her. Her left arm then began to shake. She showed her mother her arm. She was taken to the local doctor the next day and her condition diagnosed as ‘chorea’. On the way to the doctor she first noticed weakness of the left leg, it would give at the knee. She walked very badly and her mother had to support her. She came home and went to bed and has been there ever since. On admission she could not phonate, there was pain in the left shoulder, running down the left arm. Pain in the abdomen and rather coarse tremor in the forearm . . . walks with a limp . . . .’ (May G, QSR, 1917).

May was treated with psychotherapy and her symptoms were explained by ‘a history of sexual indiscretion and she has practised uncleanliness with herself’. The ‘cause of the condition’ was explained to her. May’s voice returned instantaneously, the tremor ceased for a time and ‘she was persuaded to walk without any defect’.

Some patients developed less specific symptoms of restlessness and worry following the air raids. They were, nevertheless, despite the shortages of beds, treated at the NH as inpatients, along with the soldiers with gun-shot injuries and head wounds, civilians with severe neurological conditions and soldiers with shell shock. Edith M, a 21-year-old single machinist from London, was disconcerted by the air raid of 8–9 September 1915 in central London, which had also caused some damage to the hospital:

She was very frightened at the Zeppelin raid in September 8th though the Zeppelin did not come very near her district and no one near was killed. She used to stay awake at night after this and she has not slept since. She does not think she sleeps at all throughout the night. She does not think of anything in particular but her mind wanders and is never still. She feels nervous in the night as well as the day. Her head is numbed at the back and she has queer sensations in it. She does not take any interest in reading and does not seem to enjoy anything. She feels somehow different from what she used to be . . . . (Edith M, QSR, 1916)

This case shows how air raids transformed people’s lives, deprived them of their sleep and paralysed their minds. Edith needed some respite and reassurance. Her stay at the NH over Christmas and the New Year, the sleeping medication and the head massages she received on a regular basis helped her to overcome her state of depression. Dr Adrian, the responsible junior doctor who in 1932 would receive the Nobel Prize in Physiology or Medicine for his work on the physiology of nerve cells, noted on the case record that Edith was discharged as ‘cured’.

Many patients with air-raid shock were admitted to the NH long after the war had ended, still suffering from functional symptoms that had been triggered by air raids. One of them was Sabrina R, a 20-year-old single woman from London who was treated from 10 November 1922 to 4 February 1923. ‘The right leg won’t go where I want it to go and jumps about’ was her presenting complaint, in the patient’s own words, as documented in the record. The young woman also complained of double vision, which led to the initial diagnosis of ‘disseminated sclerosis’. The following history was taken from the patient:

Patient states she has always been a nervous subject and was often spoken to at school because her hands shook. Nervous breakdown in January 1915 (aged 13). Was shaken up at the time of air raid in June 1915. Never felt well after a second nervous shock due to air raid over the Mint in June 1917. In November 1917 she experienced coldness of the tips of her fingers and toes. 14 days later had an attack of hysteria, laughing and crying for no reason. Two days after this she suddenly became unable to walk properly. She improved a little but air raids at this time always put her back again. Spent 3 months in Bart’s [St Bartholomew’s Hospital in London] in winter 1917–18 . . . . (Sabrina R, QSR, 1923)

Sabrina R showed a ‘constant rhythmic movement of her body and head’. She had to do daily exercises, received physiotherapy and sedative medication, and was eventually discharged ‘improved’.

Men also suffered long-term psychological consequences of air raids, for example Herbert P, a 35-year-old married provision dealer from London who was admitted to the NH on 8 January 1920 with a diagnosis of ‘hysteria with fugues’. His symptoms – ‘headaches and lassitude’ – had started gradually ‘during a succession of air raids’. The history is documented in the case record as follows:

Patient was in the special constabulary 2 years ago and went through several air raids all right and seemed to be getting accustomed to them. After a raid in September 1917 the legs gradually got weak and patient felt very slack. He kept to work till Xmas 1917 and then had to give it up. Patient was then very depressed, felt ‘lifeless’, suffered from dull headaches. Vision was also sometimes blurred and occasionally patient had palpitations. Patient has been ‘up and down’ all the time, never getting quite well and able to do good business. Appetite has been very variable and bowels constipated . . . . (Herbert P, QSR, 1920)

Herbert P was discharged ‘relieved’ after 23 days.

### Hospital treatment

The average time that patients with air-raid shock spent in hospital was about two months (58.6 [±36.4] days); this was similar to that established for other civilian and military admissions with functional disorders. The treatments are listed in Table 1. The majority of patients (*n* = 30; 51.7%) received physical therapies such as baths, massages and heat therapy. Sixteen patients (27.6%) were only prescribed sedatives, such as bromide salts. Other common treatments were electrotherapy (*n* = 15; 25.9%) and exercises (*n* = 12; 20.7%). Treatment outcomes were documented on the cover of the medical record by the RMO. The majority of patients with air-raid shock were classified as ‘improved’ on discharge (*n* = 35; 60.3%), seventeen (29.3%) were discharged ‘in status quo’ and only seven patients were ‘cured’ (12.1%).

### The diagnoses

Physicians documented diagnoses on the cover page of the medical record. About a quarter of patients (*n* = 15; 25.9%) with air-raid shock received an ‘organic’ diagnosis, even if there was no evidence for an organic problem. The most common ‘organic’ diagnoses given to patients with air-raid shock were ‘disseminated sclerosis’ (*n* = 8) and ‘epilepsy’ (*n* = 3). Although these neurological conditions appeared on the cover sheet of many records as official diagnoses, the history and physical examination did not confirm an organic condition but clearly pointed towards a functional somatic disorder. The second most common diagnostic group was neurasthenia (*n* = 14; 24.1%), followed by psychiatric conditions, such as ‘depression’ or ‘dementia praecox’ (*n* = 11; 19.0%).

### The symptoms

The most common functional symptoms in patients who were affected by the air raids were hyperkinetic movements, such as shaking and tremor, occurring in half of the cases (*n* = 29). Other motor symptoms such as weakness and paralysis also occurred frequently, as did somatic symptoms such as headache, dizziness and gastrointestinal complaints (*n* = 25; 43.1%); see Tables 2 and 3.

**Table 2.** Symptoms of civilians with air-raid shock (*N* = 58).

Motor symptoms:	
Hyperkinetic movements	29 (50.0%)
Weakness/paralysis	25 (43.1%)
Visual disturbances	2 (3.4%)
Auditory disturbances	5 (8.6%)
Somato-sensory disturbances	10 (17.2%)
Speech disturbances	7 (12.1%)
Fits	8 (13.7%)
Anxiety/mood disorders	15 (25.9%)
Pain*	16 (27.6%)
Catatonia	1 (1.7%)
Somatic symptoms**	25 (43.1%)
Psychotic symptoms	6 (10.3%)
Strange bodily sensations	6 (10.3%)
Dissociative states	0
Phobias	5 (8.6%)
Obsessions/compulsions	0

* Without headache (headache documented under somatic symptoms).

** See Table 3.

**Table 3.** Somatic symptom categories for patients who suffered from somatic symptoms (*N* = 25).

Gastro-intestinal*	7
Headache	14
Dizziness	7
Chest pain	6
Fatigue	7
Weakness	3
Fainting	3
Genito-urinary**	3

* e.g. diarrhoea, vomiting, nausea.

** e.g. urgency, frequency of micturition.

## Shell-shocked soldiers and air raids

To be sure, air raids traumatized not only civilians but also soldiers, who were affected in two different ways. First, air raids were just another source of worry for those men who had been called up for service (Grayzel, 2012: 76–7, 82). Soldiers – unable to protect their families at home – were concerned about the safety of their wives and children. Furthermore, the air raids were a potent trigger for soldiers to develop ‘shell shock’. Soldiers were exposed to air raids at the Front, but also when on home leave from active service. Indeed, not all soldiers who developed shell shock during World War I had been involved in active combat. Some of them developed ‘shell shock’ before being sent to the front line, or when on leave from front line service (Linden and Jones, 2014). The symptoms could be triggered by minor accidents – such as tripping on the stairs – and also by explosions in munitions factories or air raids (Linden, 2017). Soldiers with these histories were treated at the NH in London along with the physically injured and those traumatized in battle. Similar to soldiers traumatized at the front line, these men were physically unscathed.

Publications in medical journals of the time report shell-shocked soldiers whose symptoms deteriorated following air raids in France. In London, some previously cured soldiers relapsed:

. . . during the air raids [they] are nearly frantic with terror, numbers of them writhing under their beds or falling back into their former palsied condition, their eyes transfixed and their twitching faces wearing a look of abject horror. It is pitiful to hear their repeated appeals: Why can’t I go into the country, somewhere where it is quiet and restful? Is a man who has gone through what we had at the front to be kept in this hell until he loses his reason? (Lumsden, 1917)

Therefore, some doctors made an urgent plea to send traumatized soldiers to country houses rather than exposing ‘nerve-shattered men’ to air raids in the capital (p. 707). Arthur Hurst, the leading British shell shock doctor of the time, also recommended that all men suffering from war neuroses should be removed from London (Hurst and Symns, 1918).

The harmful effects of air raids on soldiers recovering from shell shock were also discussed in Parliament:

Early in November a question was asked in the House of Commons with regard to shell shock patients at the 4th London General Hospital, and other similar cases at Golder’s Green, the suggestion being that the condition of these men is aggravated by air raids and that they ought to be removed to a quieter neighbourhood . . . it is bad treatment to congregate nerve-shattered men in hospitals unless the severity of their symptoms renders this unavoidable, and that it is unpardonable to keep even the serious cases in a town like London, where air raids are frequent . . . . There can be little doubt that shell shock patients are extremely liable to relapse under the mental stress of an air raid, and when cases of varying degrees of severity are grouped together at such a time the good effects of months of treatment may be undone in the course of a few minutes. (Anon., 1918b)

## Comparing the shell-shocked soldier to the air-raid-shocked civilian

In narratives of both traumatized soldiers and civilians, found in the medical case records of the NH in London, the loss of control and unpredictability of war are two major themes of psychological breakdown. Soldiers in the trenches had to endure ‘the noises made by shells and the uncertainty of where they would strike’ (Steward B, QSR, 1915). Because of the uncertainty and the continuous threat to which they were exposed, many escaped into an alternative dream world – an altered state of consciousness which made the situation bearable (Linden, 2017). A similar experience of not being in control and not knowing what to expect was described by some of the civilians who ultimately succumbed to the new threat from the air. A witness of the air raid on London on 8–9 September 1917 reported: ‘Whole nights could be spent sleepless, sobbing and trembling, expecting a bomb to burst overhead at any moment, . . . no one and nowhere was safe’ (Fegan, 2002: 44). Like the soldiers at the Western Front, one of civilians’ greatest worries was being buried alive (p. 59).

However, unlike the soldiers at the Western Front, civilians could not take direct action against the enemy, and this might have made the feeling of not being in control even worse (Miller, 1943: 3). On 13 June 1917, the war poet Siegfried Sassoon witnessed one of the worst bombings of London while at Liverpool Street Station waiting for a train to Cambridge. Sassoon, an officer in the Royal Welch Fusiliers who had been involved in active combat at the Western Front, compared the situation of the soldier at the Western Front to that of the civilian exposed to air raids. To him, the situation at home was even more challenging than fighting in the trenches. Whereas soldiers were prepared to die, and able to actively fight the enemy, civilians remained helpless and were never prepared for the invisible enemy:

It was impossible to deny that the War was being brought home to me. . . . This sort of danger seemed to demand a quality of courage dissimilar to front line fortitude. In a trench one was acclimatized to the notion of being exterminated and there was a sense of organized retaliation. But here one was helpless; an invisible enemy sent destruction spinning down from a fine weather sky . . . . (Sassoon, 1930)

Comradeship and group cohesion were also important for boosting and keeping up morale in the troops. The medical profession realized that this resilience factor had to be reinforced for civilian populations as well, in particular in view of a future war. Civilians, like soldiers, had to be ‘well and resolutely led’; they needed to be part of a group, take responsibility for each other and fight for a common purpose (Rickman, 1939).

Even after the first air raids on Britain in World War I, there remained much uncertainty about potential future threats. Peel (1929: 148) described how ‘At one time there was a fear that gas bombs would be dropped, and almost at once gas masks of all kinds (most of which would have been quite useless) were on sale, and rapidly bought up, and gas-mask drill became a feature of family routine.’

## Children and air raids

Among the patients with air-raid shock treated at the NH were seven children. They had not been evacuated from London, like so many others. On 10 December 1915, *The Times* published a detailed article about children’s perceptions of air raids and their reactions to this new threat (Anon., 1915). It contained a report of a lecture given by Charles William Kimmins, educational psychologist and Chief Inspector of Schools for the London County Council, to the Child Study Society at the Royal Sanitary Institute on 9 December 1915. This lecture was based on 945 essays by girls and boys aged 8–13 who attended schools in areas of London that had been affected by the Zeppelin raids of 8 September and 13 October. The essays had been written 10–14 days after the air-raid experience, and the children had not been prepared for this task, but were given 15 minutes to write down their thoughts and experiences. Kimmins divided the children into different age groups and analysed responses of girls and boys separately. He observed that the youngest children of the study, boys and girls aged 8, expressed ‘no personal feelings’ and that there was ‘no evidence of fear’. Girls started becoming fearful at the age of 9, whereas the boys ‘thoroughly enjoyed the raids’ and spent as much time as possible in the streets. Boys started revealing signs of fear at the age of 10, however ‘not nearly as marked as in the case of the girls’. According to Kimmins’s observations, from the age of 11 both boys and girls had lost all sense of fear again. Whereas boys predominantly expressed curiosity and excitement when hunting for souvenirs, girls developed more nuanced emotional reactions to the air raids. Feelings of anger and calls for retaliation, as well as reflections on the justification of war and its needless suffering, are revealed in the girls’ essays, for example:

I know what our brave soldiers and sailors have had to go through day after day. This kind of thing makes one realize what war is; and yet dropping bombs on harmless people is not war. That night I felt bitter towards the Germans. I felt I could fly to Germany and do the same thing to them. (Anon., 1915)

Girls in particular took care of younger children from a very early age. Kimmins noted that in 95 per cent of the essays no reference was made to the father, and if the father was mentioned, he was depicted as being terrified by the air raids, hiding from the bombs or resorting to alcohol. Kimmins praised the resilience and determination of ‘this philosophic youth’.

The very few publications of the time in which children’s reactions to air raids are mentioned seem to confirm this notion that children were unfazed by this new experience. The official conclusion of the medical world was that ‘fears with regard to the harmful influence of air raids upon the nervous system of children have, fortunately, proved to be groundless’ (Anon., 1918a).

However, the resilience of children in the face of air raids may not have been as strong and pervasive as transpired from the essays analysed by Kimmins. During the war years, the Children’s Fresh Air Mission was set up with the purpose of ‘sending away children suffering from air raid shock’ (Anon., 1918c). Moreover, the cases of the NH demonstrate the potential long-term consequences of air raids for the mental health of children. The story of Mary K, ‘a bright little girl of 8’, whose father had been drafted into the army, is a case in point. Mary was admitted to the NH on 25 March 1918. She had been ‘shaking down the left side’ for the past two years:

The trouble started with a fit after the second Zeppelin raid. The mother could not give much detail about the fit as she saw it towards the close. The child did not cry or struggle. The next thing the mother noticed was shaking of the left hand and at the same time the child dragged the left leg. The shaking has got worse and now involves the whole of the left side. Shaking is always present, it is intensified during excitement and during air raids. She has always been bright mentally and good tempered. (Mary K, QSR, 1918)

Mary had been an inpatient in another London hospital a year before being admitted to the NH. The referral letter which was attached to the case record stated:

This child, Mary K, has suffered from ‘weakness of the left side with twitching’ since a Zepp raid about 2 yrs ago. The onset was sudden but unaccompanied by fever or a stroke . . . . Diagnosis on her in-patient paper was ?functional chorea. The movements appear to me to be entirely different from chorea; they are spasmodic rather than fidgety, and on attempting voluntary movement are wildly ataxic. (Mary K, QSR, 1918)

Mary stayed at the NH for five and a half weeks; she was prescribed general massages and a normal diet. The treatment did not improve her symptoms; she was eventually discharged ‘in status quo’.

A similar case was that of Martin G, a 12-year-old ‘delicate, thin, pale’ boy from London, who was treated at the NH with a diagnosis of ‘hysteria’. Martin had already suffered from ‘faints’ and ‘convulsions’ between the ages of five and eight. Yealland, who was in charge of the patient, noted that ‘the Zeppelin raids have accentuated [Martin’s] condition’. Martin had not walked since the Silvertown explosion in West Ham, Essex^7^ in the early evening of 19 January 1917, which had occurred just four days before his admission to hospital. This massive explosion at a munition’s factory caused substantial damage in the local area, killing 73 people and injuring 400. Yealland documented in the notes:

. . . if left alone patient gets very frightened. He has sensations of heat in the abdomen. His heart seems to stop beating and he feels a stabbing pain in the ear, throat and mouth. He has severe headache, becomes blind. There is a peculiar smell in the nostrils, like coal smoking. The ears ring. When coming to he feels as if his head were split open, he is unable to speak for a while and feels tired out. He thinks a fright is the cause of his seizures. (Martin G, QSR, 1917)

Martin had a ‘fear of walking’ and of being alone. He initially had several fits during his hospital stay. These were ‘preceded by a cry, usually he calls “nurse, nurse, come quickly”. Turns over on his right side, moans, his back becomes rigid and his head is retracted. His arms are extended and thrown upwards and the face is pale . . .’. A handwritten note by Yealland before Martin’s discharge says:

Patient very much improved. Has not had a ‘fit’ for a month. He is decidedly hysterical. When told to get out of bed he said he could not walk. After a few feeble attempts he succeeded in walking. He was easily persuaded [underscored] to walk very well. On discharge he was fairly well nourished, walked well, but the fear of being left alone persisted. (Martin G, QSR, 1917)

The effects of air raids on children in World War I, and the measures taken to protect them from potential psychological consequences, have been surprisingly little investigated. This certainly remains a topic for future research.

## The aftermath and long-term consequences

The experience of civilians being targeted by the war machine became a focal point in post-war memoirs and fiction. The potential for destruction and devastation mesmerized the crowds and provided anxious visions of a future war.

During the war, much had been published about the psychological reactions to combat trauma in soldiers, and also – to a lesser degree – about civilian stress reactions. The NH dealt with cases of air-raid shock, and indeed many narratives of other patients – with neurological conditions or severe injuries – made mention of the air raids and their transformative effect on people’s lives.

After World War I, everyone wanted to forget about the psychiatric casualties of war and a whole ‘generation of medical men [had] arisen to whom war neurosis and psychiatric casualties [were] largely unknown’ (Wright, 1939: 576). Cases of air-raid shock requiring hospital treatment after the war were ‘re-labelled’ as neurological conditions and became unrecognizable in the hospital statistics. However, at the dawn of a new conflict in 1939, the government and medical authorities resumed the discussion on psychological consequences of warfare for civilian populations, and measures to protect them. The medical profession reflected on the experiences of the previous war, and thought about preventive methods and their role in the moral reinforcement of the civilian population (*British Medical Journal*, 1939).^8^

They were right to do so; just one year after the beginning of World War II, air raids would erupt on Britain on a scale unimaginable in 1918. The link between air raids and psychological casualties would once again become a subject of intense discussion (Jones, 2016).

## Notes

1 Report of a mother with air-raid shock killing her child. Grayzel sees the air raid as a ‘convincing excuse’ rather than a plausible explanation for the mother’s ‘total breakdown’ and the killing of her child.

2 They were less likely to be detected not only because they were smaller, but also because they did not require wireless navigation.

3 The numbers of casualties are slightly different in various sources. However, this was undoubtedly the worst attack of the war, with the highest numbers of casualties.

4 One female patient who was admitted to and treated at the NH also developed severe symptoms after an air-raid warning in London.

5 All things unknown are taken to be grand.

6 As cited in: Grayzel, 1999: 45–6.

7 Now part of the London Borough of Newham, in Greater London.

8 This book was published one month before the beginning of World War II and had obviously been in preparation for some time before that.
